# Facts from Text—Is Text Mining Ready to Deliver?

**DOI:** 10.1371/journal.pbio.0030065

**Published:** 2005-02-15

**Authors:** Dietrich Rebholz-Schuhmann, Harald Kirsch, Francisco Couto

## Abstract

The mining of information from scientific literature using computational tools has tremendous potential for knowledge discovery, but how close are we to realizing this potential?

Biological databases offer access to formalized facts about many aspects of biology—genes and gene products, protein structure, metabolic pathways, diseases, organisms, and so on. These databases are becoming increasingly important to researchers. The information that populates databases is generated by research teams and is usually published in peer-reviewed journals. As part of the publication process, some authors deposit data into a database but, more often, it is extracted from the published literature and deposited into the databases by human curators, a painstaking process.

Research literature and scientific databases fulfil different needs. Literature provides ideas and new hypotheses, but is not constrained to provide facts in formats suitable for use in databases. By contrast, databases efficiently provide large quantities of data and information in a standardised schema representing a predefined interpretation of the data. While the acceptance of a paper can enforce the submission of data to a central data repository, such as EMBL (www.ebi.ac.uk/embl/) or ArrayExpress (www.ebi.ac.uk/arrayexpress/), nobody receives credit for the submission of a fact to a database without an associated publication. As long as this practice continues, curation will be necessary to add the (re)formalised facts to biological databases.

Given that publications are not about to be replaced with routine deposition of data into databases, is it possible to develop software tools to support the work of the curator? Could we automatically analyse new scientific publications routinely to extract facts, which could then be inserted into scientific databases? Could we tag gene and protein names, as well as other terms in the document, so that they are easier to recognise? How can we use controlled vocabularies and ontologies to identify biological concepts and phenomena? Fortunately, there are many groups that are now seeking to answer these questions, precisely with a view to extracting facts from text.

Part of the motivation for this effort in text mining technology is the inexorable rise in the amount of published literature (Figure 1). This massive growth, coupled with the current inefficiencies in transferring facts into other data resources, leads to the unfortunate state that biological databases tend to be incomplete (for example, DNA sequences without known function in genetic databases), and there are inconsistencies between databases and literature.

**Figure 1:**
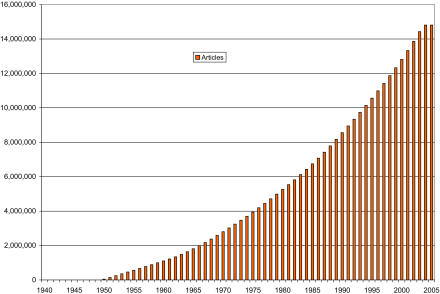
Medline Article Deluge This figure shows the exploding number of articles available from Medline over the past 65 years (data retrieved from the SRS server at the European Bioinformatics Institute; www.ebi.ac.uk/). In 2003, about 560,000 articles were added to Medline, and from 2000 to 2003, 2 million articles. (Articles already registered for 2005 are given as well.)

In theory, text mining is the perfect solution to transforming factual knowledge from publications into database entries. But computational linguists have not yet developed tools that can analyse more than 30% of English sentences correctly and transform them into a structured formal representation [1,2]. We can analyse part of a sentence, such as a subphrase describing a protein–protein interaction or part of a sentence containing a gene and a protein name, but we always run into Zipf's law whenever we write down the rules for how the extraction is done (Figure 2) [3]. A small number of patterns describe a reasonable portion of protein–protein interactions, gene names, or mutations, but many of those entities are described by a pattern of words that's only ever used once. Even if we could collect them all—which is impossible—we can't stop new phrases from being used.

**Figure 2:**
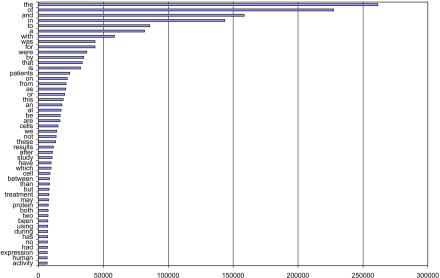
Zipf's Law Zipf's eponymous law is illustrated by the analysis of 30,000 Medline abstracts (4,952,878 occurrences of words; 144,841 different words). Frequent terms account for a large portion of the text, but a large fraction of terms appear at a low frequency and often only once (69,782 words appear only once). Zipf was a linguistic professor at Harvard University [3].

## Curators—The Gold Standard

Hand-curated data is precise, because the curator is trained to inspect literature and databases, select only high-quality data, and reformat the facts according to the schema of the database. In addition, curators select citations from the text as evidence for the identified fact, and those citations are also added to the database.

Curators read and interpret the text at the same time, and if they don't understand the meaning of a sentence, they can go back and pick a new strategy to analyse it—they can even call the authors to iron out any ambiguities. Curators can also cope with the high variability of language described by Zipf's law. At present, no computer-based system comes close to matching these capabilities. In particular, it is difficult to convert all the curators' domain knowledge into a structured training set for the purposes of machine learning approaches.

Curators fulfil a second important task: they know how to define standards for data consistency, in particular, the most relevant terminology, which has led to the design of standardised ontologies and controlled vocabularies (see Box 1 for an explanation of these and related terms). Examples of these include Gene Ontology (GO; www.geneontology.org/), Unified Medical Language System (www.nlm.nih.gov/research/umls/), and MedDRA (www.meddramsso.com/NewWeb2003/index.htm) [4]. These terminological resources help to relate entries in bioinformatics databases to concepts mentioned in scientific publications and to link related information in databases using different schemas. Text miners would love such standards to be used in text, but there is an understandable reluctance to impose and use standards that might limit the expressiveness of natural language.

**Box 1. Glossary**

**Controlled vocabulary:** A set of terms, to standardise input to a database.

**F-measure:** A statistic that is used to score the success of NE recognition by text mining tools. The F-measure is an average parameter based on precision (how many of the entities found by the tool are correct identifications of an entity) and recall (how many of the entities existing in the text did the tool find).

**Machine learning:** The technology and study of algorithms through which machines (computers) can “learn”, or automatically improve their systems through data gathered in the past (experience).

**Ontology:** A set of terms with clear semantics (language), clear motivations for distinction between the terms, and strict rules for how the terms relate to each other.

## Curation and Text Mining—In Partnership

The problem with curation of data is that it is time consuming and costly, and therefore has to focus on the most relevant facts. This compromises the completeness of the curated data, and curation teams are doomed to stay behind the latest publications. So, is it possible for curation and text mining to work together for rapid retrieval and analysis of facts with precise postprocessing and standardisation of the extracted information?

There are several software tools that perform well in the identification of standardised terms from the literature. Examples include Textpresso and Whatizit [5,6,7,8]. Extensive term lists come from the Human Genome Organization (www.gene.ucl.ac.uk/hugo; 20,000 gene and protein names), GO (almost 20,000 terms), Uniprot/Swiss-Prot (www.ebi.uniprot.org/index.shtml; about 200,000 terms), and other databases. In addition, terms describing diseases, syndromes, and drugs are available from the Unified Medical Language System. Altogether, about 500,000 terms constitute the basis of domain knowledge in life sciences. To gain some perspective of this figure: an average individual handles 2,000 to 20,000 terms in his or her daily language, and *Merriam-Webster's Collegiate Dictionary* provides definitions for 225,000 terms (www.merriam-webstercollegiate.com/).

**Figure 3:**
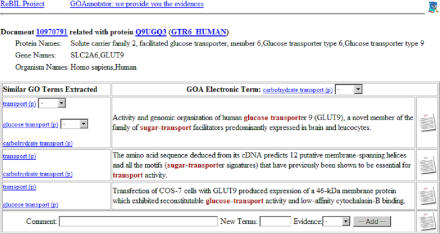
GOAnnotator The illustrated software tool brings together data from text mining and from databases to support curators in the GO annotation of proteins (Couto FM, Lee V, Dimmer E, Camon E, Apweiler R, et al., unpublished data). Here a protein is shown in conjunction with the GO terms that have been gathered from various databases and attributed to the protein through electronic annotation. Both are evaluated against similar GO terms extracted from text documents. The curator looks into the evidence and decides whether any of the GO terms extracted from the documents should be assigned to the protein.

The identification of all terms by a text mining system still sets challenging demands. All variants of a term have to be taken into account, including syntactical variants and synonyms. In the case of ambiguities, relevant findings have to be distinguished from other findings—a process referred to as disambiguation. Depending on the curation task, it might therefore be advantageous to select only part of the terminological resources and thus restrict the domain of the terminology to the curators' needs (Figure 3).

Available text mining solutions are concerned with named entity (NE) recognition (entities are, for example, proteins, species, and cell lines), with identification of relationships between NEs (such as protein interactions), and with the classification of text subphrases according to annotation schemata in general (thyroid receptor is a thyroid hormone receptor) [9,10,11,12,13,14,15]. Whilst the identification of a curation team's terminology in the scientific text under scrutiny is immensely valuable, there is still a long way to go before this becomes routine.

## Some Immediate Challenges

Not all terms used in the literature (NEs) can actually be found in some kind of database (perhaps because of an author error, or an alternative name for an entity adopted by the community). Text mining methods therefore have to detect new terms and map the term to known terminology [16]. If several mappings are possible, the correct version has to be selected (disambiguation).

Over the past several years text mining research teams have presented various approaches that train a software tool to locate representations of gene or protein names (for example, BioCreative, www.pdg.cnb.uam.es/BioLINK/BioCreative.eval.html, and JNLPBA, www.genisis.ch/~natlang/JNLPBA04/) [17,18]. These tools are scored with a statistic known as the F-measure, with the best methods scoring about 0.85. At the level of 0.85, curators still tend to be unhappy. However, analyses have shown that this score is in the range of curator–curator variation (unpublished data, measured as part of the project work for [19]), which suggests that such methods produce useful results.

Additional information-extraction methods have been proposed, for example, for the documentation of mutations in specific genes and for the extraction of the subcellular location of proteins [11,13]. An even larger number of tools focus on the identification of appropriate terminology for the annotation of genes (GO terms) [7]. The evaluation of their usefulness depends on the demands of the user groups. Finally, another way to support curation teams would be to provide information-retrieval methods to guide the team members towards documents containing relevant information. For example, in 2002, the participants in the Knowledge Discovery and Data-Mining Challenge Cup (www.cs.cornell.edu/projects/kddcup/) had to select documents from a given corpus that contained relevant experimental results about Drosophila [20].

## How Can Publishers Contribute?

For all automated information-extraction methods, it is obvious that access to literature is crucial. Electronic access has, of course, already had a huge impact, but the structure and organisation of manuscripts could also be improved. For example, semantic tags could be integrated into the text. The markup would not appear on web pages or when the document is printed, but it would help software to deal with semantic aspects of the document. Inserting tags, for example, to mark protein names would allow retrieval software to find documents about proteins even if they look like common English words, such as “you” or “and”. Retrieval engines currently often ignore such terms. In addition, explicit tags would enable text mining methods, for example, when looking for protein–protein interactions, to use the correct semantic interpretation.

Text mining systems already available today, such as Whatizit, can integrate semantic tags during submission, which have to be verified by the author. Text mining is ready to deliver tools whereby information is passed back to the authors about the proper use of terminology within their documents. If the use of a term raises conflicts or ambiguities or if the use of a term is wrong, the author is asked to provide feedback. The curation effort is resolved at the earliest possible time-point. Author, publisher, reviewer, and reader profit from consistent information representation, which leads to better dissemination of documents and journals and easily offsets the additional cost in the generation of an article. Publishers and authors have to agree on standards though.

## Is Text Mining Ready to Deliver?

Text mining solutions have found their way into daily work, wherever fast and precise extraction of details from a large volume of text is needed. We have to keep in mind, however, that any text mining tool, just like other bioinformatics resources, will only be suitable for a limited number of tasks. For example, the same text may serve curators from different communities who extract different types of facts, depending on their domain knowledge. Furthermore, different communities have different expectations for accuracy. For example, curators dealing with a small set of proteins prefer tools with high recall, whereas curators dealing with a large number of proteins prefer tools with high precision.

Although text mining cannot dissect English sentences completely, and cannot extract the meaning and put the facts into a database, text mining tools are becoming increasingly used and valued. Text mining is ready to deliver handling of complex terminology and nomenclature as a mature service. It is only a matter of time and effort before we are able to extract facts automatically. The consequences are likely to be profound. Not only will we have a more effective approach for the mining of knowledge from the literature, our approach to the publication process itself might change. If a fact is clear enough for automatic extraction, it could be reported in a fact database instead of a publication. As methods improve, authors will see more and more of their text being analysed and formalised in a database. If appropriate quality control is provided, and if authors receive due credit for their deposition of facts into databases, we might well see a shift towards original papers describing new creative ideas and visions rather than just listing facts.

## Abbreviations

GO: Gene Ontology
NE: named entity
